# Involvement of older people in the development of fall detection systems: a scoping review

**DOI:** 10.1186/s12877-016-0216-3

**Published:** 2016-02-11

**Authors:** Friederike JS Thilo, Barbara Hürlimann, Sabine Hahn, Selina Bilger, Jos MGA Schols, Ruud JG Halfens

**Affiliations:** Applied Research & Development in Nursing, Health Division, Bern University of Applied Sciences, Murtenstrasse 10, CH-3008 Bern, Switzerland; School CAPHRI, Department of Health Services Research, Maastricht University, Maastricht, The Netherlands; School CAPHRI, Department of General Practice, Maastricht University, Maastricht, The Netherlands; Departments of Pulmonary Medicine and Thoracic Surgery, Inselspital, Bern University Hospital, Bern, Switzerland

**Keywords:** Fall detection, User-involvement, Scoping review, Older people

## Abstract

**Background:**

The involvement of users is recommended in the development of health related technologies, in order to address their needs and preferences and to improve the daily usage of these technologies.

The objective of this literature review was to identify the nature and extent of research involving older people in the development of fall detection systems.

**Methods:**

A scoping review according to the framework of Arksey and O’Malley was carried out. A key term search was employed in eight relevant databases. Included articles were summarized using a predetermined charting form and subsequently thematically analysed.

**Results:**

A total of 53 articles was included. In 49 of the 53 articles, older people were involved in the design and/or testing stages, and in 4 of 53 articles, they were involved in the conceptual or market deployment stages. In 38 of the 53 articles, the main focus of the involvement of older people was technical aspects. In 15 of the 53 articles, the perspectives of the elderly related to the fall detection system under development were determined using focus groups, single interviews or questionnaires.

**Conclusions:**

Until presently, involvement of older people in the development of fall detection systems has focused mainly on technical aspects. Little attention has been given to the specific needs and views of older people in the context of fall detection system development and usage.

**Electronic supplementary material:**

The online version of this article (doi:10.1186/s12877-016-0216-3) contains supplementary material, which is available to authorized users.

## Background

People aged 65 years and older are the age group mostly affected by falls and their subsequent negative health consequences [1–3]. Approximately 25 to 35 % of them have had one or more falls per year [2, 4]. A fall is defined as “an unexpected event in which the participants come to rest on the ground, floor, or lower level” [5]. Falls significantly affect mortality, morbidity and quality of life as well as health care costs among the aging population [1, 6–11]. Falls are also one of the main causes of physical injuries [4, 12, 13], are a frequent causal factor for hospital admissions [14] and are additionally a precipitator for institutional long-term care admissions [15, 16]. The rates of falls and the outlined associated negative consequences are twice as high for persons over 75 years of age [17].

One determining factor influencing the severity of fall consequences in older people is the amount of time spent lying on the floor or ground [18, 19]. This is particularly critical when a person cannot call for help, for instance when she/he has lost consciousness or is alone when the fall occurred. Even when uninjured, 47 % of people who have experienced a fall were unable to get up after without help [20]. Lying on the floor due to a fall event for one hour or more is defined as a “long-lie” [21]. Experiencing a “long-lie” event is associated with serious injuries, higher mortality rates and hospital admissions, as well as consequent care home admissions [18, 19, 22]. 13 % [21] to 20 % [23] of older people admitted to a hospital due to a fall have experienced a “long-lie” event. In order to avoid this and ensure prompt assistance, early fall detection is vital among community-dwelling older people. A fall detection system could be defined as a system which detects falls and alerts a designated person or emergency services, in order to facilitate rapid assistance [24, 25]. A fall detection system could prevent or limit impairment and subsequently allow preservation of activities of daily living. Although the literature outlines a variety of fall detection systems [25, 26], minimal and faulty use of fall detection systems in community-dwelling older people has been reported [18, 27–32]. Hence, it seems that the target users, older people, struggle with the usability of available fall detection systems in their daily lives [18, 27, 32].

In order to provide fall detection systems which meet the needs and expectations of older people, user involvement should be applied to the different stages of development of the system, as is described with other health-related technologies [33].

The process of user involvement is relevant because it facilitates the development of technology that is «need-driven» and «not technology-driven» [34]. It enables a better understanding of the process of the interaction and the surrounding context between technology and its users [35, 36]. User involvement may help to ensure that preferences are considered from the beginning of the development process [33, 37, 38], and may therefore improve the level of acceptance of the users [39]. It might facilitate short- and long-term usage and hence decrease costs and the need for redesign at a later time [38]. Additionally, it might increase sales, decrease training costs and decrease user support [39].

Currently, an overview of the nature and extent of published research regarding the involvement of older people in the development, testing and/or evaluation of fall detection systems is lacking. Recent literature reviews have focused on the technical aspects of available fall detection systems or on the views of older people, carers and health professionals regarding benefits or challenges of fall detection systems and aspects related to their implementation [24–26, 40, 41].

Therefore, the aim of this scoping review is to identify the state of research involving older people in the development of fall detection systems. The research question is: What is the nature and extent of user involvement of people 65 years of age and older, in the development, testing and/or evaluation of fall detection systems?

## Methods

To answer the research question a scoping review was undertaken. A scoping review is a particularly suitable type of literature review for gaining a comprehensive overview of the research field of interest [42, 43]. A scoping review differs from a systematic review in that a scoping review does not aim to assess the effectiveness of an intervention; instead it aims to assess the feasibility of a systematic review and to guide for future research. A scoping review also aims to map the nature and extent of research activities and provides a rigorous and transparent methodology [43]. It provides a descriptive overview of the analysed articles without critically appraising the quality of the included studies [44]. Five stages are characteristic for a scoping review [42, 43]. Stage one refers to the identification of the research question (see above). Stages two to five comprise the process of the literature search and conclude with the reporting of the results. They were carried out as follows:

### Stage 2: Identifying relevant studies

A systematic literature search was conducted between May and July 2014 across nursing, medicine and engineering disciplines in the following eight databases: IEEE Xplore@ Digital Library, Inspec, Scopus, Compendex, BIOSIS Previews, Cochrane Library, CINAHL/EBESCO and PubMed. A key word strategy was applied, which was developed progressively by two members of the research team (FJST and SB) and was approved by a third member of the research team (SH). The following terms were used: fall detection, fall, aged, old, senior, elder*, monitoring, device, system, sensor, fall risk assessment, fall prevention, gait assessment. These terms were combined with the Boolean operators AND, OR, or NOT. An additional search of reference lists of articles fulfilling the inclusion criteria was carried out.

### Stage 3: Study selection

Literature published in English, French or German within the last ten years (2004–2014) was retrieved in order to reflect the recent research trends. The time limit was chosen due to the visible evolvement of technology during this period. Moreover, according to the literature, there is an evolving body of knowledge regarding user-involvement in the development of health-related technologies in the last ten years. Articles were eligible for inclusion if they: 1) focused on either development, testing and/or evaluation regarding a fall detection system; 2) included in their study at least one older person who was involved in the development or who was a target person to test or to evaluate the system; 3) the older person was 65 years of age and older, or was defined as “older”. Records were identified, duplicates removed, titles screened, abstracts examined and the remaining full-texts assessed for eligibility. The assessment of titles and abstracts was carried out independently by two reviewers (FJST, BH). There were few discrepancies. The first reviewer assessed all full-text articles for eligibility and 20 % of the retained full-text articles were assessed by the second reviewer independently. The first reviewer (FJST) randomly selected the full-text articles for the second reviewer (BH). Differences in the article screening and selection process were solved by consensus. Only one article was discussed with a third reviewer (SB) due to a lack of consensus concerning eligibility.

Currently, reporting guidelines for scoping reviews do not exist [44]. Therefore, in accordance to the recommendation of Pham et al. [44], the Preferred Reporting Items for Systematic Reviews and Meta-Analysis [45] was used to report the flow of articles from identification to final inclusion.

### Stage 4: Charting the data

A data charting form was iteratively developed focusing on themes relevant to the research question and was based on the following: author (s), year, title, country, publication type, design, aim, type of fall detection system, fall detection alert, placement of system, methodology (narrative description of user involvement in the development/testing/evaluation), sample size, sample characteristics and setting. The first draft of the data charting form was tested independently on 20 % of a second random reselection of articles by two researchers (FJST, BH). After critical comparison of these results, discrepancies were resolved through discussion. Subsequently, the revised data charting form was critically discussed and jointly approved by the research team. The charting form was then applied to all included articles in order to narratively extract the data. In studies containing a mixed population (participants under 65 years as well as 65 years and older), only data related to older people was extracted, and only with those who involved a target person in the development, testing and/or evaluation of a fall detection system.

### Stage 5: Collating, summarizing and reporting the results

The charted findings were numerically and thematically [46] analysed and then summarized from a descriptive perspective. Absolute frequencies were calculated for the numerical description of the nature and extend of literature. The following themes were identified, describing the nature (A.-B.) and extend (C.) of the literature:

A. *General aspects of literature involving older people*: Author (s) year, country, design, type of fall detection system (wearable system - e.g. body-worn sensors; or environmental systems - e.g. infrared sensors or camera) fall detection alert, sample size, mean age in years, gender of involved older people and length of test time.
B. *Descriptive characteristics of the involved older people*: age gender, height and/or weight and/or BMI, state of health, fall risk (no risk, at risk, at high risk), fear of falling and fall history (with, without).
C. Focus involvement and stage (s) of involvement of older people:

Focus of involvement:

i. Involvement of older people for technical aspects such as simulation or performance of either *scripted activities of daily living* (*ADL*), which signifies that study participants had to perform a series of ADL (sitting down or walking), or of *everyday life activities*, which signifies that study participants had to perform as usual their activities of daily living during the testing stage of a fall detection system.
ii. Involvement of older people to investigate their views on fall detection systems.

Stage (s) of involvement

ᅟ The framework of user involvement in the development of medical device technology according to Shah et al. [33] was used to define the stages of user involvement in each article:
I Idea generation and concept development
II Device (re-) design and prototype development
III Prototype testing involving in-house and trials in the real field
IV Device deployment in the market and user feedback

In order to test the process of stage five, 20 % of the articles from a third random selection were analysed and summarized independently by the first and second reviewer (FJST, BH). After critical comparison of these results, discrepancies were resolved through discussion. Afterwards, all included articles were analysed and summarized by the first reviewer. This result was subsequently critically discussed and jointly approved by the research team.

This scoping review is part of a research project, which was approved by the Ethical Committee of the Canton of Bern (Z020/2014).

## Results

**Fig. 1:**
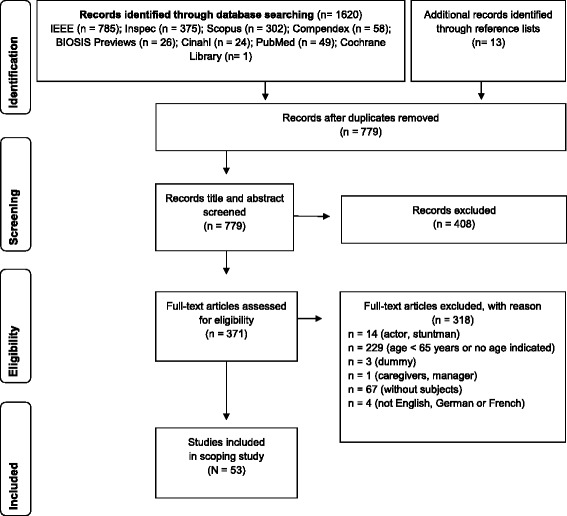
Flow chart of study selection [45]

After the selection process, as displayed in Fig. 1, a total of 53 studies was included from the original 1633 potential relevant records [47–99].

The findings relevant to the research question are summarized in the following three tables: nature of literature - Additional file 1: Table S1 and Additional file 2: Table S2 and extent of literature - Additional file 3: Table S3.

A. General aspects of the literature
  Additional file 1: Table S1 presents the general aspects of the analysed literature involving older people in the development, testing and/or evaluation of fall detection systems. Researchers from four continents have carried out studies involving older people in the development, testing and/or evaluation of fall detection systems, although most of this research has been conducted in Europe (see Additional file 1: Table S1). 37 studies focused on wearable systems, ten on environmental systems, four on both and two on fall detection systems amongst other technologies (e.g. general event reminder or monitoring of physiological parameters) (see Additional file 1: Table S1).
  The sample size in the included studies varied. In more than three-fourths (n = 47) of the studies, the sample size ranged from one to 35 older people. In five studies the sample size was not clearly stated and in one study it included a sample of 97 older people.
  The length of test time in which older people were involved in the respective studies was reported in 20 articles, and it ranged from 40 minutes to several hours, days, weeks until 1.5 years (see Additional file 1: Table S1). In nearly half of the studies (n = 25), it was not stated and in eight studies this criterion was not applicable due to the study design.
B. Descriptive characteristics of the involved older people
  Information regarding the age of the involved older people was reported in 45 studies (see Additional file 2: Table S2). Eight studies characterised their sample by describing them as old or elderly people. 27 articles displayed information about the gender of the study participants (see Additional file 2: Table S2). In nine studies the involved older people were described as healthy. In 14 studies fall risk, fear of falling and/or fall history was assessed (see Additional file 2: Table S2).
C. Focus and stage (s) of involvement
  Additional file 3: Table S3 shows the findings regarding the focus of involvement, distinguishing between the technical aspects and the views of the older people as well as defining the stage (s) of involvement of the older people.

### Focus of involvement

i. Technical aspects
  Nearly three-fourths (n = 38) of the included studies solely examined the technical aspects of fall detection systems, without involving the views of older persons (see Additional file 3: Table S3). Five studies investigated both the technical aspects and the views of the older people in the context of fall detection systems.
  Regarding the settings of involvement of older people, most studies were (n = 12) carried out in the home environment, followed by the nursing home and the hospital setting (see Additional file 3: Table S3). Five studies reported the laboratory environment as the setting of involvement for older people. In three studies, older people were involved in more than one setting and eleven studies did not clearly state the setting of involvement (see Additional file 3: Table S3).
  During their involvement, in order to develop a valid and reliable fall detection algorithm, older people were invited to simulate *scripted ADL* and/or to perform *everyday life activities* (see method section). Most frequently (n = 22) they simulated *scripted ADL* and in six studies older people simulated both *scripted ADL* and performed *everyday life activities* (see Additional file 3: Table S3).
ii. Views of older people
  Ten studies focused solely on the views of older persons related to the fall detection system under development. Aspects of their examinations were, for example, usability, perceptions of usefulness, concerns associated with usage, attractiveness or wearing comfort. Their views were investigated in 15 studies utilizing focus group interviews, single interviews and/or questionnaires. The focus group interviews were carried out both with and without a fall detection system prototype and both with and without visual material showing fall detection systems or fall scenarios (see Additional file 3: Table S3).

### Stage (s) of involvement

Older people were involved in all four of the following stages of development and testing of a fall detection system: In three studies - stage I, in 18 studies - stage II, in 28 studies - stage III and in one study - stage IV. In three studies, older people were involved in two stages; stages II and III (see Additional file 3: Table S3). According to the analysed literature, there was no study reporting on the development of a fall detection system which involved older people in three of the four stages, nor in all four stages.

A comparison of the included studies showed that six research groups [51–58, 73, 74, 76, 77, 94, 95, 97, 98] published several articles referring to results either from stage II or stage III of development without reporting on the examination of the view of involved older people regarding the fall detection system under development (see Additional file 3: Table S3). Quagliarella et al. [86, 87] published two articles referring to results from the same fall detection system; once from stage II and once from stage III, however without reporting on the examination of the view of the involved older people.

## Discussion

This scoping review shows that older people are predominantly involved in the design and testing stages of fall detection systems under development, with a strong focus on the technical aspects. Most of this research was carried out in Europe with the majority of the studies targeting the development of wearable fall detection systems. Information regarding the older people involved was in general limited to age and gender.

This scant description of the study participants is noteworthy, given that older people are a heterogeneous age group [100]. In regards to fall detection systems, characteristics of the target users, such as risk of falling, history of falls or fear of falling, might influence the needs and expectations of older people [101]. Moreover, the functional and cognitive status of older people might affect their handling and, therefore, their acceptance of the technology. Aging is linked to a change of cognitive abilities and cognitive impairment may hinder the use of a fall detection system [102]. Considering the cognitive abilities of the target group might enhance the development of a fall detection system which is also easy for people with an impaired cognitive status to learn and to use. On the other hand, it might be considered that carers of cognitively impaired people should also be involved in the development of a fall detection system.

However, it seems that the scant description is in accordance with the predominant involvement of older people in the technical aspects of the design and testing stages. It might be argued that for the technical aspects of a fall detection system under development, study participants’ information such as age, gender or BMI is sufficient. Nevertheless, Shah and Robinson [103] indicated that specific information concerning the targeted users is relevant, because different users employ the same technology in different settings and contexts. This argument is confirmed by Kaufman et al. [104] in their evaluation of a telemedicine system. They reported that even if users are a part of the same setting or age group, they differ considerably in terms of knowledge, competencies, need for social help, health status or self-efficacy.

This scoping review also revealed that if older people were involved, it occurred mostly in their home setting, which is congruent with the idea that the development and validation of fall detection systems should be carried out with the target group in their «real-world», and not only in laboratory settings [25, 40]. Reliable and valid fall detection and alert systems are a very important aspect. Several researchers stressed that developing a fall detection system based on falls from the real-world might minimize the rate of false positive and false negative alarms [105, 106]. Consequently, the involvement of older people in stages two and/or three, as well as in their real-world setting, seems to be very important in ensuring valid and reliable fall detection systems. Nevertheless, user involvement primarily aims to investigate needs and requirements promoting the daily use of fall detection systems [25, 35].

This scoping review also revealed that older people’s views regarding fall detection systems were scarcely used. The reasons for this could be the higher costs or difficulties in accessing the targeted users [39]. Shah et al. [38] specified that minimal user involvement might originate from issues in confidentiality, (e.g. patent application) in terms of bringing technology into the market or due to limited experience of user involvement in the research group. Moreover, knowing the views (e.g., needs or preferences) of users might be challenging for a research team. According to the view of users, the design of a product or a system should then be adapted [39]. However, there could be gap between the users’ views and the technical, product or design solutions [34]. The disaccord is that user involvement might be challenging and depends upon research resources. Hence, it seems important to carry out user involvement if there are «real» possibilities for users in influencing design or solutions of a fall detection system. If not, it is conceivable that user involvement might generate legitimate cost concerns, as well create frustration with the involved users due to their views not being considered in the further development of the fall detection system.

Older people were involved in all four stages of fall detection system development. However, their involvement was mostly limited to the design and testing stages. As discussed, the older target users were hardly involved in the beginning and end stages of the development process. Additionally, older people were minimally involved in several other stages of development and no article described involvement exceeding two stages of development. Hence, due to the predominant involvement in the prototype design and testing stages, it is logical that technical aspects of the fall detection system under development are at the forefront. However, the involvement of the target users in all stages of development is associated with several advantages [38, 39]. Involvement of target users beginning in the first stage fosters their influence in the concept development of the fall detection system and thus avoids additional cost in later stages due to redesign issues. User involvement from the first stage onward may improve the level of acceptance of fall detection systems by older people due to the early focus on their views (e.g., needs, preferences or requirements) including the practical aspects of daily living. Furthermore, it could be considered that user feedback also in the last stage is important in order to identify any aspects that are lacking. These aspects may not be completely related to technical issues and it is conceivable that support or coaching services, e.g. from health care providers, would help to enable older people in long-term usage of fall detection systems in daily life. Moreover, the fourth stage may provide ideas, due to recurrent needs of target users, for the further development of fall detection systems such as combining them with additional features (e.g. capturing mobility patterns or physiological parameters). This would signify that the involved older people would «truly» have the possibility to enhance the meaningful use of fall detection system in daily life.

The literature scrutinized in this scoping review illustrated that there is not yet widespread involvement of older people from the beginning until the last stage of development. It also revealed that it is not yet common practice to investigate older peoples’ views regarding fall detection systems under development. User involvement can be interpreted in different ways. According to Kujala [107] and Damodoran [108], the user may be involved for providing information (informative), for comments about a predefined matter (consultative) or for influencing decision making regarding a system under development (participative). It seems that apart from the stages and methods of involvement, it should also be considered whether users be involved in an informative, consultative or a participative way. In the context of fall detection systems, the participative way would best facilitate older people to influence the decision making process as it necessitates capturing their views, needs, preferences, requirements or issues of the practical usage of fall detection systems in daily life.

### Strengths and limitations

This scoping review applied a rigorous and transparent methodology throughout the five recommended steps of the framework from Arksey and O’Malley [43]. In order to ensure that the research question was explored in breadth, literature was searched in eight data bases across different disciplines. Although relevant published articles may have been omitted, for instance, due to the limitation of articles written in English, German and French, references from the selected articles did not indicate any other important studies.

Scoping reviews have been criticised for lacking methodological details during stage five, with *collating*, *summarizing and reporting the results* of the framework [109]. Therefore, this scoping review transparently described, utilizing the thematical analysis approach [46], how stage five was conducted.

The validation of the applied framework of Shah et al. [33], describing the four stages of health-related technology development is still missing, according to the authors. However, due to the health related context of technologies as well the clear description of each stage, this framework was considered and shown to be suitable for the underlying study.

## Conclusions

This scoping review reveals that older people were predominantly involved in the *design and testing of* fall detection systems, which denotes that the focus was on the technical aspects in the development of fall detection algorithms. In the development and use of fall detection systems, little attention has been given to the views, needs, preferences or practical aspects of usefulness in daily life of the older people themselves. This may be an important factor in explaining, in general, the minimal and often faulty use of fall detection systems in the daily lives of older people.

A more «need-driven» rather than «technology-driven» approach in the development of fall detection systems is necessary. Therefore, future research also needs to involve older people more in *idea generation and concept development* as well as in *device deployment and market and user feedback*. Future involvement should consider a more participative way to capture the views, needs, preferences, requirements or issues for the practical use of fall detection systems in the daily lives of older people. This may positively influence the daily usage and acceptance of fall detection systems in community-dwelling older people.

## Additional files

Additional file 1: Table S1. General aspects of literature involving older people in the development of fall detection systems (PDF 259 kb) Additional file 2: Table S2. Descriptive characteristics of the older people involved in the development of fall detection systems (PDF 170 kb) Additional file 3: Table S3. Focus and stage (s) of involvement of older people in the development of fall detection systems (PDF 238 kb)

## Abbreviations

ADL: activities of daily living

## References

[1] Centers for Disease Control and Prevention. Falls Among Older Adults: An Overview. 2012. http://www.cdc.gov/homeandrecreationalsafety/falls/adultfalls.html. Accessed 23 Aug 2013.

[2] BFS. Gehvermögen und Stürze. Bundesamt für Statistik. 2013. http://www.bfs.admin.ch/bfs/portal/de/index/themen/14/02/01/key/07/05.html. Accessed 23 Aug 2013.

[3] Meyer K (2009). Gesundheit in der Schweiz - Nationaler Gesundheitsbericht 2008.

[4] WHO. Global report on falls prevention in older age. 2007. Available from: http://www.who.int/ageing/publications/Falls_prevention7March.pdf. Accessed: 10 Apr 2014.

[5] Lamb SE, Jorstad-Stein EC, Hauer K, Becker C (2005). Development of a common outcome data set for fall injury prevention trials: the Prevention of Falls Network Europe consensus. J Am Geriatr Soc.

[6] Hanley A, Silke C, Murphy J (2011). Community-based health efforts for the prevention of falls in the elderly. Clin Interv Aging.

[7] Gründler BM. Sturzprävention für Senioren und Seniorinnen. 2006. Bern. Schweizerische Beratungsstelle für Unfallverhütung.

[8] Hester AL, Wei F (2013). Falls in the community: state of the science. Clin Interv Aging.

[9] Stenhagen M, Ekstrom H, Nordell E, Elmstahl S (2014). Accidental falls, health-related quality of life and life satisfaction: a prospective study of the general elderly population. Arch Gerontol Geriatr.

[10] Tuunainen E, Rasku J, Jantti P, Pyykko I (2014). Risk factors of falls in community dwelling active elderly. Auris Nasus Larynx.

[11] Sartini M, Cristina ML, Spagnolo AM, Cremonesi P, Costaguta C, Monacelli F (2010). The epidemiology of domestic injurious falls in a community dwelling elderly population: an outgrowing economic burden. Eur J Public Health.

[12] Scuffham P, Chaplin S, Legood R (2003). Incidence and costs of unintentional falls in older people in the United Kingdom. J Epidemiol Community Health.

[13] LPZ Maastricht (2011). Instruktionsmaterial und Begleitdokumente LPZ, Messzyklus 2011.

[14] Canadian Institute for Health Information. National Trauma Registry 2011 Report: Hospitalizations for Major Injury in Canada, 2008–2009 Data. 2011. Available from: https://secure.cihi.ca/free_products/NTR_CDS_2008_2009_Annual_Report.pdf. Accessed: 10 Apr 2014.

[15] Department of Health. National Service Framework for Older People. 2001. Available from: https://www.gov.uk/government/uploads/system/uploads/attachment_data/file/198033/National_Service_Framework_for_Older_People.pdf. Accessed: 10 Apr 2014.

[16] Todd C, Skelton D (2004). What are the main risk factors for falls amongst older people and what are the most effective interventions to prevent these falls?.

[17] Rubenstein LZ. Falls in older people: epidemiology, risk factors and strategies for prevention. Age Ageing. 2006;35 Suppl 2:ii37-ii41. doi:10.1093/ageing/afl084. 10.1093/ageing/afl084 16926202

[18] Fleming J, Brayne C, Cambridge City over-75 s Cohort study c. Inability to get up after falling, subsequent time on floor, and summoning help: prospective cohort study in people over 90. BMJ. 2008;337:a2227. doi:10.1136/bmj.a2227. 10.1136/bmj.a2227 PMC2590903 19015185

[19] Lord SR, Sherrington C, Menz HB (2001). Falls in oder people. Risk, factors and strategies for prevention.

[20] Tinetti ME, Liu WL, Claus EB (1993). Predictors and prognosis of inability to get up after falls among elderly persons. JAMA.

[21] Simpson PM, Bendall JC, Tiedemann A, Lord SR, Close JC (2014). Epidemiology of emergency medical service responses to older people who have fallen: a prospective cohort study. Prehosp Emerg Care.

[22] Ryynanen OP, Kivela SL, Honkanen R, Laippala P (1992). Falls and lying helpless in the elderly. Z Gerontol.

[23] Vellas B, Cayla F, Bocquet H, De Pemille F, Albarede JL (1987). Prospective study of restriction of activity in old people after falls. Age Ageing.

[24] Igual R, Medrano C, Plaza I (2013). Challenges, issues and trends in fall detection systems. Biomedical engineering online.

[25] Chaudhuri S, Thompson H, Demiris G (2014). Fall Detection Devices and Their Use With Older Adults: A Systematic Review. J Geriatr Phys Ther.

[26] Ward G, Holliday N, Fielden S, Williams S (2012). Fall detectors: a review of literature. J Assist Technol.

[27] Johnston K, Grimmer-Somers K, Sutherland M (2010). Perspectives on use of personal alarms by older fallers. International journal of general medicine.

[28] Johnston K, Worley A, Grimmer-Somers K, Sutherland M, Amos L (2010). Personal alarm use to call the ambulance after a fall in older people: characteristics of clients and falls. Journal of Emergency Primary Health Care (JEPHC).

[29] Mann WC, Belchior P, Tomita MR, Kemp BJ (2005). Use of personal emergency response systems by older individuals with disabilities. Assist Technol.

[30] Heinbuchner B, Hautzinger M, Becker C, Pfeiffer K (2010). Satisfaction and use of personal emergency response systems. Z Gerontol Geriatr.

[31] Nyman SR, Victor CR. Use of personal call alarms among community-dwelling older people. Ageing & Society. 2014;34 (1):67–89. doi:Doi10.1017/S0144686x12000803.

[32] Zingaro S (2012). Nottelefone teils untauglich und überteuert.

[33] Shah SGS, Robinson I, AlShawi S. Developing medical device technologies from users’ perspectives: A theoretical framework for involving users in the development process. International journal of technology assessment in health care. 2009;25 (4):514–21. doi:Doi10.1017/S0266462309990328. 10.1017/S0266462309990328 19845981

[34] Bridgelal Ram M, Grocott P, Weir H (2007). Issues and challenges of involving users in medical device development. Health expectations : an international journal of public participation in health care and health policy.

[35] Rodeschini G (2011). Gerotechnology: a new kind of care for aging? An analysis of the relationship between older people and technology. Nurs Health Sci.

[36] Gulliksen J, Goransson B, Boivie I, Blomkvist S, Persson J, Cajander A. Key principles for user-centred systems design. Behav Inform Technol. 2003;22 (6):397–409. doi:Doi10.1080/01449290310001624329.

[37] De Vito DA, Myers BA, Mc Curry KR, Dunbar-Jacob J, Hawkins RP, Begey A (2009). User-centered design and interactive health technologies for patients. Computers, informatics, nursing : CIN.

[38] Shah SG, Robinson I (2007). Benefits of and barriers to involving users in medical device technology development and evaluation. Int J Technol Assess Health Care.

[39] Kujala S. User involvement: a review of the benefits and challenges. Behav Inform Technol. 2003;22 (1):1–16. doi:Doi10.1080/0144929021000055530.

[40] Schwickert L, Becker C, Lindemann U, Marechal C, Bourke A, Chiari L (2013). Fall detection with body-worn sensors A systematic review. Zeitschrift fuer Gerontologie und Geriatrie.

[41] Hawley-Hague H, Boulton E, Hall A, Pfeiffer K, Todd C (2014). Older adults’ perceptions of technologies aimed at falls prevention, detection or monitoring: A systematic review. Int J Med Inform.

[42] Levac D, Colquhoun H, O’Brien KK. Scoping studies: advancing the methodology. Implement Sci. 2010;5. doi:Doi10.1186/1748-5908-5-69. 10.1186/1748-5908-5-69 PMC2954944 20854677

[43] Arksey H, O’Malley L (2005). Scoping studies: towards a methodological framework. Int J Soc Res Methodol.

[44] Pham MT, Rajic A, Greig JD, Sargeant JM, Papadopoulos A, McEwen SA (2014). A scoping review of scoping reviews: advancing the approach and enhancing the consistency. Health expectations : an international journal of public participation in health care and health policy.

[45] Moher D, Liberati A, Tetzlaff J, Altman DG, Group P (2009). Preferred reporting items for systematic reviews and meta-analyses: the PRISMA statement. J Clin Epidemiol.

[46] Dixon-Woods M, Agarwal S, Jones D, Young B, Sutton A (2005). Synthesising qualitative and quantitative evidence: a review of possible methods. Journal of health services research & policy.

[47] Abbate S, Avvenuti M, Bonatesta F, Cola G, Corsini P, Vecchio A. A smartphone-based fall detection system. Pervasive Mob Comput. 2012;8 (6):883–99. doi:DOI10.1016/j.pmcj.2012.08.003.

[48] Ariani A, Redmond SJ, Chang D, Lovell NH (2010). Software simulation of unobtrusive falls detection at night-time using passive infrared and pressure mat sensor.

[49] Barralon P, Dorronsoro I, Hernandez E. Automatic fall detection: Complementary devices for a better fall monitoring coverage. 2013 IEEE 15th International Conference on e-Health Networking, Applications and Services (Healthcom 2013). 2013:590–3. doi:10.1109/HealthCom.2013.6720745.

[50] Bloch F, Gautier V, Noury N, Lundy JE, Poujaud J, Claessens YE (2011). Evaluation under real-life conditions of a stand-alone fall detector for the elderly subjects. Annals of physical and rehabilitation medicine.

[51] Bourke AK, Lyons GM (2008). A threshold-based fall-detection algorithm using a bi-axial gyroscope sensor. Med Eng Phys.

[52] Bourke AK, O’Brien JV, Lyons GM. Evaluation of a threshold-based tri-axial accelerometer fall detection algorithm. Gait & Posture. 2007;26 (2):194–9. doi:DOI10.1016/j.gaitpost.2006.09.012. 10.1016/j.gaitpost.2006.09.012 17101272

[53] Bourke AK, Prescher S, Koehler F, Cionca V, Tavares C, Gomis S (2012). Embedded fall and activity monitoring for a wearable ambient assisted living solution for older adults. Engineering in Medicine and Biology Society (EMBC), 2012 Annual International Conference of the IEEE; 2012 Aug. 28 2012-Sept.

[54] Bourke AK, Van De Ven P, Gamble M, O’Connor R, Murphy K, Bogan E (2010). Applications of waist segment kinematic measurement using accelerometry for an autonomous fall-detection system during continuous activities.

[55] Bourke AK, Van De Ven P, Gamble M, O’Connor R, Murphy K, Bogan E (2010). Assessment of waist-worn tri-axial accelerometer based fall-detection algorithms using continuous unsupervised activities.

[56] Bourke AK, van de Ven P, Gamble M, O’Connor R, Murphy K, Bogan E et al. Evaluation of waist-mounted tri-axial accelerometer based fall-detection algorithms during scripted and continuous unscripted activities. J Biomech. 2010;43 (15):3051–7. doi:10.1016/j.jbiomech.2010.07.005. 10.1016/j.jbiomech.2010.07.005 20926081

[57] Bourke AK, Van De Ven PWJ, Chaya A, Olaighin G, Nelson J (2008). Design and test of a long-term fall detection system incorporated into a custom vest for the elderly.

[58] Bourke AK, Van De Ven PWJ, Chaya AE, OLaighin GM, Nelson J (2008). Testing of a long-term fall detection system incorporated into a custom vest for the elderly.

[59] Boyle J, Karunanithi M (2008). Simulated fall detection via accelerometers.

[60] Campo E, Bonhomme S, Chan M, Esteve D. Remote tracking patients in retirement home using wireless multisensor system. 2010 12th IEEE International Conference on e-Health Networking, Applications and Services (Healthcom 2010). 2010:226–30. doi:10.1109/health.2010.5556567.

[61] Che-Chang Y, Yeh-Liang H (2007). Algorithm Design for Real-time Physical Activity Identification with Accelerometry Measurement. Industrial Electronics Society, 2007. IECON 2007. 33rd Annual Conference of the IEEE; 2007 5–8 Nov.

[62] De La Guia Solaz M, Bourke A, Conway R, Nelson J, Olaighin G, De La Guia Solaz M, Bourke A, Conway R, Nelson J, Olaighin G (2010). Real-time low-energy fall detection algorithm with a Programmable Truncated MAC.

[63] Demiris G, Rantz MJ, Aud MA, Marek KD, Tyrer HW, Skubic M (2004). Older adults’ attitudes towards and perceptions of ‘smart home’ technologies: a pilot study. Medical Informatics and the Internet in Medicine.

[64] Fourty N, Guiraud D, Fraisse P, Perolle G, Etxeberria I, Val T (2009). Embedded system used for classifying motor activities of elderly and disabled people. Comput Ind Eng.

[65] Gietzelt M, Spehr J, Ehmen Y, Wegel S, Feldwieser F, Meis M (2012). GAL@Home A feasibility study of sensor-based in-home fall detection. Zeitschrift fuer Gerontologie und Geriatrie.

[66] Godfrey A, Bourke AK, Olaighin GM, van de Ven P, Nelson J. Activity classification using a single chest mounted tri-axial accelerometer. Med Eng Phys. 2011;33 (9):1127–35. doi: DOI 10.1016/j.medengphy.2011.05.002. 10.1016/j.medengphy.2011.05.002 21636308

[67] Goevercin M, Koeltzsch Y, Meis M, Wegel S, Gietzelt M, Spehr J (2010). Defining the user requirements for wearable and optical fall prediction and fall detection devices for home use. Inform Health Soc Ca.

[68] Holzinger A, Searle G, Pruckner S, Steinbach-Nordmann S, Kleinberger T, Hirt E et al. Perceived usefulness among elderly people: Experiences and lessons learned during the evaluation of a wrist device. 2010 4th International Conference on Pervasive Computing Technologies for Healthcare (Pervasive Health 2010). 2010:5 pp.- pp. doi:10.4108/icst.pervasivehealth2010.8912.

[69] Horton K. Falls in older people: The place of telemonitoring in rehabilitation. Journal of Rehabilitation Research and Development. 2008;45 (8):1183–94. doi:Doi10.1682/Jrrd.2007.09.0152. 19235119

[70] Huang CN, Chiang CY, Chiu CB, Hsu SJ, Chan CT (2012). Accelerometry-based motion pattern analysis for reliable fall detection. Adv Sci Lett.

[71] Jantaraprim P, Phukpattaranont P, Limsakul C, Wongkittisuksa B (2012). A system for improving fall detection performance using critical phase fall signal and a neural network. Songklanakarin J Sci Technol.

[72] John SG, Owen PJ, Smith K, Youde JH, McIntyre CW (2008). Utilisation of telemedicine to assess energy expenditure and stability in older people with chronic kidney disease. Computers in Cardiology, 2008; 2008 14–17 Sept.

[73] Kangas M, Vikman I, Nyberg L, Korpelainen R, Lindblom J, Jamsa T. Comparison of real-life accidental falls in older people with experimental falls in middle-aged test subjects. Gait & Posture. 2012;35 (3):500–5. doi:DOI10.1016/j.gaitpost.2011.11.016. 10.1016/j.gaitpost.2011.11.016 22169389

[74] Kangas M, Vikman I, Wiklander J, Lindgren P, Nyberg L, Jamsa T (2009). Sensitivity and specificity of fall detection in people aged 40 years and over. Gait Posture.

[75] Kerdegari H, Samsudin K, Ramli AR, Mokaram S. Evaluation of fall detection classification approaches. Proceedings of the 2012 4th International Conference on Intelligent & Advanced Systems (ICIAS). 2012:131–6. doi:10.1109/icias.2012.6306174.

[76] Lai CF, Huang YM, Park JH, Chao HC (2010). Adaptive Body Posture Analysis for Elderly-Falling Detection with Multisensors. Ieee Intelligent Systems.

[77] Lai C-F, Sung-Yen C, Han-Chieh C, Yueh-Min H (2011). Detection of Cognitive Injured Body Region Using Multiple Triaxial Accelerometers for Elderly Falling. Sensors Journal, IEEE.

[78] Lindemann U, Hock A, Stuber M, Keck W, Becker C (2005). Evaluation of a fall detector based on accelerometers: A pilot study. Med Biol Eng Comput.

[79] Liu J, Lockhart TE (2013). Automatic individual calibration in fall detection an integrative ambulatory measurement framework. Computer methods in biomechanics and biomedical engineering.

[80] Londei ST, Rousseau J, Ducharme F, St-Arnaud A, Meunier J, Saint-Arnaud J (2009). An intelligent videomonitoring system for fall detection at home: perceptions of elderly people. J Telemed Telecare.

[81] Marquis-Faulkes∗ F, McKenna SJ, Newell AF, Gregor P (2005). Gathering the requirements for a fall monitor using drama and video with older people. Technol Disabil.

[82] McKenna SJ, Marquis-Faulkes F, Newell AF, Gregor P (2006). Requirements gathering using drama for computer vision-based monitoring in supportive home environments. Gerontechnology.

[83] Miao Y, Yuanzhang Y, Rhuma A, Naqvi SMR, Liang W, Chambers JA (2013). An online one class support vector machine-based person-specific fall detection system for monitoring an elderly individual in a room environment. IEEE journal of biomedical and health informatics.

[84] Narasimhan R (2012). Skin-contact sensor for automatic fall detection. Conf Proc IEEE Eng Med Biol Soc.

[85] Parker S, Nussbaum G, Sonntag H, Pühretmair F, Williams V, McCrindle R (2008). ENABLE - A view on user’s needs. 11th International Conference on Computers Helping People with Special Needs, ICCHP 2008. Linz.

[86] Quagliarella L, Sasanelli N, Belgiovine G. An interactive fall and loss of consciousness detector system. Gait & Posture. 2008;28 (4):699–702. doi:DOI10.1016/j.gaitpost.2008.04.011. 10.1016/j.gaitpost.2008.04.011 18657976

[87] Quagliarella L, Sasanelli N, Belgiovine G (2008). Performances of an accelerometric device for detecting fall and loss of consciousness. J Appl Biomater Biom.

[88] Rantz MJ, Skubic M, Abbott C, Galambos C, Pak Y, Ho DKC et al. In-Home Fall Risk Assessment and Detection Sensor System. J Gerontol Nurs. 2013;39 (7):18–22. doi:Doi10.3928/00989134-20130503-01. 10.3928/00989134-20130503-01 PMC3817841 23675644

[89] Shinmoto Torres RL, Ranasinghe DC, Qinfeng S, Sample AP (2013). Sensor enabled wearable RFID technology for mitigating the risk of falls near beds. RFID (RFID), 2013 IEEE International Conference on; 2013 April 30 2013-May 2.

[90] Sixsmith A, Johnson N. A smart sensor to detect the falls of the elderly. Ieee Pervas Comput. 2004;3 (2):42–7. doi:Doi10.1109/Mprv.2004.1316817.

[91] Soaz C, Lederer C, Daumer M (2012). A new method to estimate the real upper limit of the false alarm rate in a 3 accelerometry-based fall detector for the elderly.

[92] Stone E, Skubic M. Fall Detection in Homes of Older Adults Using the Microsoft Kinect. Biomedical and Health Informatics, IEEE Journal of. 2014;PP (99):1-. doi:10.1109/JBHI.2014.2312180. 10.1109/JBHI.2014.2312180 24733032

[93] Tamrat T, Griffin M, Rupcic S, Kachnowski S, Taylor T, Barfield J. Operationalizing a wireless wearable fall detection sensor for older adults. 2012 6th International Conference on Pervasive Computing Technologies for Healthcare. 2012:297–302. doi:10.4108/icst.pervasivehealth.2012.248643.

[94] van de Ven P, Bourke AK, Nelson J, Laighin GO (2008). A wireless platform for fall and mobility monitoring. IET Irish Signals and Systems Conference ISSC.

[95] van de Ven P, Feld R, Bourke A, Nelson J, Laighin GO (2008). An integrated fall and mobility sensor and wireless health signs monitoring system. IEEE Sensors.

[96] Wang J, Zhang Z, Li B, Lee S, Sherratt RS (2014). An enhanced fall detection system for elderly person monitoring using consumer home networks. Consumer Electronics, IEEE Transactions on.

[97] Wu G, Xue S (2010). Automatic fall detection based on kinematic characteristics during the pre-impact phase of falls. 6th World Congress of Biomechanics, WCB 2010. Conjunction with 14th International Conference on Biomedical Engineering.

[98] Wu G, Xue SW. Portable preimpact fall detector with inertial sensors. IEEE Trans Neural Syst Rehabil Eng. 2008;16 (2):178–83. doi:Doi10.1109/Tnsre.2007.916282. 10.1109/TNSRE.2007.916282 18403286

[99] Zhang T, Wang J, Xu L, Liu P, Huang DS, Li K, Irwin GW (2006). Fall detection by wearable sensor and one-class SVM algorithm. Lecture Notes in Control and Information Sciences.

[100] Grigsby JS (1996). The meaning of heterogeneity: An introduction. Gerontologist.

[101] Kim J, Park HA. Development of a Health Information Technology Acceptance Model Using Consumers’ Health Behavior Intention. Journal of Medical Internet Research. 2012;14 (5). doi:DOI10.2196/jmir.2143. 10.2196/jmir.2143 PMC3510715 23026508

[102] Chen K, Chan A (2013). Use or Non-Use of Gerontechnology—A Qualitative Study. International Journal of Environmental Research and Public Health.

[103] Shah SGS, Robinson I (2008). Medical device technologies: who is the user. Int J Healthcare Technology and Management.

[104] Kaufman DR, Patel VL, Hilliman C, Morin PC, Pevzner J, Weinstock RS (2003). Usability in the real world: assessing medical information technologies in patients’ homes. J Biomed Inform.

[105] Klenk J, Becker C, Lieken F, Nicolai S, Maetzler W, Alt W (2011). Comparison of acceleration signals of simulated and real-world backward falls. Med Eng Phys.

[106] Kangas M, Korpelainen R, Vikman I, Nyberg L, Jämsä T (2015). Sensitivity and False Alarm Rate of a Fall Sensor in Long-Term Fall Detection in the Elderly. Gerontology.

[107] Kujala S. Effective user involvement in product development by improving the analysis of user needs. Behav Inform Technol. 2008;27 (6):457–73. doi:Doi10.1080/01449290601111051.

[108] Damodaran L. User involvement in the systems design process - A practical guide for users. Behav Inform Technol. 1996;15 (6):363–77. doi:Doi10.1080/014492996120049.

[109] Davis K, Drey N, Gould D. What are scoping studies? A review of the nursing literature. International journal of nursing studies. 2009;46 (10):1386–400. doi:DOI10.1016/j.ijnurstu.2009.02.010. 10.1016/j.ijnurstu.2009.02.010 19328488

