# Supplementary material for: Involvement of older people in the development of fall detection systems: a scoping review
Source: BMC Geriatr. 2016 Feb 11;16:42. doi: 10.1186/s12877-016-0216-3 (PMC4750302; doi:10.1186/s12877-016-0216-3)
Supplement: Additional file 1: Table S1. — General aspects of literature involving older people in the development of fall detection systems (PDF 259 kb) [file 12877_2016_216_MOESM1_ESM.pdf]

Involvement of Older People in the Development of Fall Detection Systems: A Scoping Review

Table 1: General aspects of literature involving older people in the development of fall detection systems

**Author(s) and year**	**Country**	**Design**	**Type of fall detection system**				**Fall detection alert** (yes / no)	**Involved older people**			
			Wearable	Placement	Environmental	Placement		Sample size (n)	Mean age in years (range or SD)	Gender: female (n)	Length of test time in day(s) (range)
Abbate et al (2012)	Italy	Exploratory	Sensor / Smartphone (accelerometer)	Waist / belt	N.a.	N.a.	Yes	10	N.s. *[1] (60-82)	4	N.a. *[2]
Ariani et al (2010)	Australia	Exploratory	N.a.	N.a.	Passive infrared sensors (PIRs) and Pressure mats (PMs)	PIR wall mounted and PM in front of doorway of every room; PM on bed, on chair, on the floor in front of toilet seat, shower and sofa.	Yes	10	N.s. (50-70)	5	N.s.
Barralon et al (2013)	Spain	Evaluation	Mobile Telecare device (accelerometer)	Waist	Shower Rug (pressure sensors)	Bathroom	Yes	20	N.s. (55-82)	N.s.	(approx. 7- 90)
Bloch et al (2011)	France	Evaluation	Vigi'Fall® (accelerometer)	Thorax	Infrared sensor	Room (not specified)	Yes	10	83 (7.5)	N.s.	ø = 21 (SD 19)
Bourke et al (2008a)	Ireland	Exploratory	Sensor (bi-axial gyroscope)	Trunk	N.a.	N.a.	No	10	77 (70-83)	3	N.s.
Bourke et al (2007)	Ireland	Exploratory	Sensor (tri-axial accelerometer)	Trunk & thigh	N.a.	N.a.	No	10	77 (70-83)	3	N.s.
Bourke et al (2012)	Germany, Ireland, Portugal, Spain, United Kingdom	Exploratory	Sensors (tri-axial accelerometer) & mobile phone	Chest, left under arm, right thigh	N.a.	N.a.	Yes	8	77 (5)	N.s.	N.s.
								6	77 (5)	N.s.	5
								9	69 (14)	4	28
Bourke et al	Ireland	Exploratory	Sensors / mobile	Waist	N.a.	N.a.	No	10	79 (73-	4	3-7

Author(s) and year	Country	Design	Type of fall detection system				Fall detection alert (yes / no)	Involved older people			
			Wearable	Placement	Environmental	Placement		Sample size (n)	Mean age in years (range or SD)	Gender: female (n)	Length of test time in day(s) (range)
(2010a)			phone (tri-axial accelerometer)						90)		hours per participant
Bourke et al (2010b)	Ireland	Evaluation	Sensors (tri-axial accelerometer)	Waist (belt)	N.a.	N.a.	No	10	79 (73-90)	4	<8 hours per participant
Bourke et al (2008b)	Ireland	Exploratory	Sensor (tri-axial accelerometer) & mobile phone integrated in a vest	left upper arm or chest	N.a.	N.a.	Yes	10	N.s., elderly	N.s.	24 (8 hours a day, 6 days a week)
Bourke et al (2010c)	Ireland	Evaluation	Sensor (tri-axial accelerometer)	Waist	N.a.	N.a.	No	10	79 (73-90)	4	3-7 hours per participant
Bourke et al (2008c)	Ireland	Exploratory	Sensor (tri-axial accelerometer) integrated in a vest & mobile phone	left upper arm or chest	N.a.	N.a.	Yes	10	N.s., elderly	N.s.	24 (8 hours a day, 6 days a week)
Boyle & Karunanithi (2008)	Australia	Exploratory	PAL Technologies (accelerometer)	Waist (belt)	N.a.	N.a.	No	15	67 (18)	N.s.	⌀=18 (2-81)
Campo et al (2010)	France	Exploratory	N.a.	N.a.	Infrared sensors (motion sensor network)	Placed on ceiling to focus bed and around bed, in bathroom, near chair and table	Yes	1	75 (N.s)	N.s.	N.s.
Che-Chang et al (2007)	Taiwan	Exploratory	Sensor (tri-axial accelerometer)	Waist (belt)	N.a.	N.a.	No	5	N.s., elderly	N.s	N.s
De la Guia	Ireland	Exploratory	Sensor (tri-axial	Trunk	N.a.	N.a.	No	10	77 (70-	3	N.s.

Author(s) and year	Country	Design	Type of fall detection system				Fall detection alert (yes / no)	Involved older people			
			Wearable	Placement	Environmental	Placement		Sample size (n)	Mean age in years (range or SD)	Gender: female (n)	Length of test time in day(s) (range)
Solaz et al (2010)			accelerometer)						83)		
Demiris et al (2004)	USA	Qualitative descriptive	Fall detection technologies amongst other health technologies				N.a.	15	N.s. (65<)	8	N.a.
Fourty et al (2009)	France, Spain	Exploratory	Portable system (bi-axial accelerometer)	N.s.	N.a.	N.a.	Yes	9	76 (N.s)	6	N.s.
Marquis-Faulkes & al (2005)	UK	Qualitative descriptive	N.a.	N.a.	Camera	Ceiling-mounted	Yes	31	N.s. (65<)	N.s. clea rly	N.a.
Gietzelt et al (2012)	Germany	Evaluation	Sensor (tri-axial accelerometer)	Chest	Camera	Wall-mounted	No	3	87 (81-92)	1	60 - Approx. 10h / day
Godfrey et al (2011)	UK, Ireland,	Exploratory	Sensor (tri-axial accelerometer)	Chest	N.a.	N.a.	No	10	77 (70-83)	3	N.s.
Goevercin et al (2010)	Germany	Qualitative descriptive	N.a.	N.a.	surveillance camera type, motion detector, fish-eye type camera, in-wall camera	Room: wall or in-wall mounted	No	22	70 (50-85)	16	N.a.
Holzinger et al (2010)	Germany, Austria, Switzerlan d	Evaluation	Watch (accerometer)	Wrist	N.a.	N.a.	Yes	N.s. clea rly	N.s. (55<)	N.s.	N.a.
Horton (2008)	UK	Observation al	pendant alarm or fall detector	Neck or worn on body (N.s.)	Bed occupancy sensor	Under mattress	Yes	35	78 (65<)	22	Weeks: 15 (4)
Huang et al (2012)	Taiwan	Exploratory	Sensor (tri-axial accelerometer)	Wrist	N.a.	N.a.	No	3	80 (11)	N.s.	N.s.
Jantaraprim et al (2012)	Thailand	Exploratory	Sensor (tri-axial accelerometer)	Trunk	N.a.	N.a.	No	14	68 (4)	7	N.s.

Author(s) and year	Country	Design	Type of fall detection system				Fall detection alert (yes / no)	Involved older people			
			Wearable	Placement	Environmental	Placement		Sample size (n)	Mean age in years (range or SD)	Gender: female (n)	Length of test time in day(s) (range)
Kangas et al (2012)	Finland, Sweden	Exploratory	Sensor (3D accelerometer)	Waist	N.a.	N.a.	No	16	88 (5)	13	N.s. (60-180)
Kangas et al (2009)	Finland, Sweden	Exploratory	Sensor (tri-axial accelerometer)	Waist	N.a.	N.a.	No	21	83 (9)	10	N.s.
Kerdegari et al (2012)	Malaysia	Exploratory	Sensor (accelerometer)	Waist	N.a.	N.a.	No	1	65 (N.s)	N.s.	N.s.
Lai et al (2010)	South Korea	Exploratory	Sensors (accelerometer)	Neck, waist, both wrists, both thighs	N.a.	N.a.	No	1	N.s., elderly	N.s.	N.s.
Lai et al (2011)	Taiwan	Exploratory	Sensors (tri-axial accelerometer)	Both hands, trunk, both feet	N.a.	N.a.	No	16	N.s., elderly	N.s.	N.s.
Lindemann et al (2005)	Germany	Exploratory	Sensors (accelerometer)	Head (hearing aid housing)	N.a.	N.a.	No	1	83 (N.s)	1	N.s.
Shinmoto et al (2013)	Australia, USA	Exploratory	Sensor (accelerometer)	Sternum	N.a.	N.a.	No	14	N.s. (66-86)	N.s.	N.s.
Liu & Lockhart (2013)	USA	Exploratory	Inertial measurement unit & sensor (tri-axial accelerometer	Thigh / trunk	N.a.	N.a.	No	10	75 (6)	N.s.	N.s.
Londei et al (2009)	Canada	Mixed Method	N.a.	N.a.	Intelligent videomonitoring system (IVS)	Room(s) at home (N.s.)	Yes	25	79 (65-87)	19	N.s.
McKenna et al (2006)	UK	Qualitative descriptive	N.a.	N.a.	Vision-based monitoring	N.a.	Yes	N.s. clearly	N.s (65<)	N.s.	N.a.
Yu et al (2013)	China	Exploratory	N.a.	N.a.	Camera	Room, wall-mounted close to ceiling	No	1	N.s., old person	N.s.	N.s.
Narasimhan (2012)	USA	Exploratory	Sensors (tri-axial accelerometer)	Torso	N.a.	N.a.	No	15	74 (63 – 91)	5	N.s.
Parker et al (2008)	Austria, UK, Ireland	Descriptive	Fall detection technologies amongst other health technologies				N.a.	97	76 (57-94)	65	N.a.

Author(s) and year	Country	Design	Type of fall detection system				Fall detection alert (yes / no)	Involved older people			
			Wearable	Placement	Environmental	Placement		Sample size (n)	Mean age in years (range or SD)	Gender: female (n)	Length of test time in day(s) (range)
Quagliarella et al (2008a)	Italy	Exploratory	Sensor (single-axis accelerometer)	N.s.	N.a.	N.a.	Yes	10	76 (3)	5	N.s.
Quagliarella et al. (2008b)	Italy	Exploratory	Device (triaxial transducer, data-logger SARI)	Waist	N.a.	N.a.	Yes	10	76 (3)	5	N.s.
Rantz et al (2013)	USA	Exploratory	N.a.	N.a.	Sensor system: pulse-Doppler range control radar (General Electric®), Microsoft Kinect®, two web cameras	Radar: Next front door, Kinect: above front door; Cameras, N.s.	Yes	15	57 (23-67)	8	
								17 (after one year n=11)	88 (67-98)	10	1.5 years
Sixsmith & Johnson (2004)	UK	Exploratory	N.a.	N.a.	Infrared array technology	Wall-mounted close to ceiling	Yes	28	N.s. (65-82)	N.s.	N.a.
Soaz et al (2012)	Germany	Exploratory	Actibelt® (3D accelerometer)	Waist	N.a.	N.a.	No	5	70 (N.s)	5	N.s. clearly
Stone & Skubic (2014)	USA	Evaluation	N.a.	N.a.	Microsoft Kinect® (depth imaging sensor)	Wall-mounted close to ceiling	No	16	N.s. (67-97)	9	One year
Tamrat et al (2012)	USA, India	Exploratory	Sensors (tri-axial accelerometer)	Each wrist, each hip, neck	N.a.	N.a.	No	12	79 (66-91)	64 %	6 or 12 (non-consecutive), 4.8h/ day (SD

Author(s) and year	Country	Design	Type of fall detection system				Fall detection alert (yes / no)	Involved older people			
			Wearable	Placement	Environmental	Placement		Sample size (n)	Mean age in years (range or SD)	Gender: female (n)	Length of test time in day(s) (range)
											3.2)
van de Ven et al (2008a)	Ireland	Exploratory	Sensor (tri-axial accelerometer) & mobile phone	chest	N.a.	N.a.	Yes	N.s.	N.s., elderly	N.s.	<8 hours per day for four weeks
van de Ven et al (2008b)	Ireland	Exploratory	Sensor (tri-axial accelerometer) & mobile phone	Chest or under left arm	N.a.	N.a.	Yes	N.s.	N.s., elderly	N.s.	<8 hours per day for four weeks
Wang et al (2014)	China, Korea, UK	Exploratory	Sensor (accelerometer) & mobile phone	Chest	N.a.	N.a.	Yes	N.s.	N.s. (50-70)	N.s.	Two weeks
Wu & Xue (2010)	USA	Exploratory	Sensor (tri-axial accelerometer)	Waist	N.a.	N.a.	No	14	N.s. (72-91)	N.s.	40-60 minutes
Wu & Xue (2008)	USA	Feasibility	Sensor (tri-axial accelerometer and triaxial angular rate sensor)	Waist	N.a.	N.a.	No	14	N.s. (72-91)	N.s.	N.s.
Zhang et al (2006)	China	Exploratory	Sensor (tri-axial accelerometer)	Waist	N.a.	N.a.	No	12	N.s. (10-70)	4	N.s.
John et al (2008)	UK	Exploratory	PreventFall Monitor (accelerometers)	Waist	N.a.	N.a.	No	5	N.s. (71-84)	2	263 (total)

*[1]Not stated; *[2]Not applicable
