# Supplementary material for: Involvement of older people in the development of fall detection systems: a scoping review
Source: BMC Geriatr. 2016 Feb 11;16:42. doi: 10.1186/s12877-016-0216-3 (PMC4750302; doi:10.1186/s12877-016-0216-3)
Supplement: Additional file 2: Table S2. — Descriptive characteristics of the older people involved in the development of fall detection systems (PDF 170 kb) [file 12877_2016_216_MOESM2_ESM.pdf]

## Involvement of Older People in the Development of Fall Detection Systems: A Scoping Review

**Table 2: Descriptive characteristics of the older people involved in the development of fall detection systems**

| **Characteristics (x= yes, described)** / **Author(s) (Year)** | Abbate et al (2012) | Ariani et al (2010) | Barralon et al (2013) | Bloch et al (2011) | Bourke et al (2008a) | Bourke et al (2007) | Bourke et al (2012) | Bourke et al (2010a) | Bourke et al (2010b) | Bourke et al (2008b) | Bourke et al (2010c) | Bourke et al (2008c) | Boyle & Karunanithi (2008) | Campo et al (2010) | Che-Chang et al (2007) | Demiris et al (2004) | Fourty et al (2009) | Marquis-Faulkes et al (2005) | Gietzelt et al (2012) | Godfrey et al (2011) |
|---|---|---|---|---|---|---|---|---|---|---|---|---|---|---|---|---|---|---|---|---|
| **Without characteristics** | | | | | | | | | | X | | X | | | X | | | | | |
| **Age** | X | X | X | X | X | X | X | X | X | | X | | X | X | | X | X | X | X | X |
| **Gender** | X | X | | | X | X | X | X | X | | | | | | | | X | X | X | X |
| **Height and/or Weight and/or BMI** | | X | | | | | | | X | | | | | | | | | | | |
| **Healthy** | | | | | | | | X | X | | X | | | | | | X | | | X |
| **Fall risk** | | | | | | . | | | | | | | | | | | | | | |
| No risk | | | | | | | | | | | | | | | | | | | | |
| At risk | | | | X | | | | | | | | | | | | | | | | |
| At high risk | | | | | | | | | | | | | X | | | | | | | |
| **Fear of falling (x=yes)** | | | | | | | | | | | | | | | | | | | | |
| **Fall history** | | | | | | | | | | | | | | | | | | | | |
| With | | | | | | | | | | | | | | | | | X | X | X | |
| Without | | | | | | | | | | | | | | | | | X | | | |

| **Characteristics** / **Author(s) (Year)** | Goevercin et al (2010) | Holzinger et al (2010) | Horton (2008) | Huang et al (2012) | Jantaraprim et al (2012) | Kangas et al (2012) | Kangas et al (2009) | Kerdegari et al (2012) | Lai et al (2010) | Lai et al (2011) | Lindemann et al (2005) | Shinmoto et al (2013) | Liu & Lockhart (2013) | Londei et al (2009) | McKenna et al (2006) | Yu et al (2013) | Narasimhan (2012) | Parker et al (2008) | Quagliarella et al (2008a) | Quagliarella et al (2008b) | Rantz et al (2013) |
|---|---|---|---|---|---|---|---|---|---|---|---|---|---|---|---|---|---|---|---|---|---|
| **Without characteristics** | | | | | | | | | X | X | | | | | | X | | | | | |
| **Age** | X | X | X | X | X | X | X | X | | | X | X | X | X | X | | X | X | X | X | X |
| **Gender** | X | | X | | X | X | | | | | X | | | X | | | X | X | X | X | X |
| **Height and/or Weight and/or BMI** | | | | | | | | | | | | | X | | | | X | | X | X | |
| **Healthy** | | | | X | | | | | | | X | | | | | | | | X | X | |

| Characteristics | Author(s) (Year) | Goevercin et al (2010) | Holzinger et al (2010) | Horton (2008) | Huang et al (2012) | Jantaraprim et al (2012) | Kangas et al (2012) | Kangas et al (2009) | Kerdegari et al (2012) | Lai et al (2010) | Lai et al (2011) | Lindemann et al (2005) | Shinmoto et al (2013) | Liu & Lockhart (2013) | Londei et al (2009) | McKenna et al (2006) | Yu et al (2013) | Narasimhan (2012) | Parker et al (2008) | Quagliarella et al (2008a) | Quagliarella et al (2008b) | Rantz et al (2013) |
|---|---|---|---|---|---|---|---|---|---|---|---|---|---|---|---|---|---|---|---|---|---|---|
| **Fall risk** | | | | | | | | | | | | | | | | | | | | | | X |
| No risk | | | | | | | | | | | | | | | | | | | | | | |
| At risk | | X | | X | | | | | | | | X | | | | | | | | | | |
| At high risk | | X | | | | | | | | | | | | | | | | | | | | |
| **Fear of falling (x=yes)** | | X | | | | | | | | | | | | | | | | | | | | |
| **Fall history** | | | | | | | | | | | | | | | | | | | | | | |
| With | | X | | X | | | | X | | | | | | | X | X | | | X | | | |
| Without | | X | | | | | | | | | | | | | | | | | X | | | |

| Characteristics | Author(s) (Year) | Sixsmith & Johnson (2004) | Soaz et al (2012) | Stone & Skubic (2014) | Tamrat et al (2012) | van de Ven et al (2008a) | van de Ven et al (2008b) | Wang et al (2014) | Wu & Xue (2010) | Wu & Xue (2008) | Zhang et al (2006) | John et al (2008) |
|---|---|---|---|---|---|---|---|---|---|---|---|---|
| **Without characteristics** | | | | | | X | X | | | | | |
| **Age** | | X | X | X | X | | | X | X | X | X | X |
| **Gender** | | | X | X | X | | | | | | | X |
| **Height and/or Weight and/or BMI** | | | | | | | | | | | | X |
| **Healthy** | | | | | | | | | | | | |
| **Fall risk** | | | | | | | | | | | | |
| No risk | | | | | | | | | | | | |
| At risk | | | | | | | | | | | | |
| At high risk | | | | | X | | | | | | | |
| **Fear of falling (x=yes)** | | | | | | | | | | | | |
| **Fall history** | | | | | | | | | | | | |
| With | | | | | X | | | | | | | |
| Without | | | | | | | | | | | | |
