# Supplementary material for: Involvement of older people in the development of fall detection systems: a scoping review
Source: BMC Geriatr. 2016 Feb 11;16:42. doi: 10.1186/s12877-016-0216-3 (PMC4750302; doi:10.1186/s12877-016-0216-3)
Supplement: Additional file 3: Table S3. — Focus and stage (s) of involvement of older people in the development of fall detection systems (PDF 238 kb) [file 12877_2016_216_MOESM3_ESM.pdf]

Involvement of Older People in the Development of Fall Detection Systems: A Scoping Review

Table 3: Focus and stage(s) of involvement of older people in the development of fall detection systems

**Author(s) and year**	**Involvement of older people**																			
	**Focus of involvement**																**Stages of involvement*[4]**			
	**Technical aspects**										**Views of older people**						**I**	**II**	**III**	**IV**
	Simulation / performance																			
			Setting																	
			Lab	Real-life																
	Scripted ADL*[3]	Everyday life activities		Home	Hospital	Nursing home	Community centre	Independent living facility	Long -term care facility	N.s. clearly	Focus group interview (s) WITH prototype	Focus group interview (s)	Focus group interview (s) WITH visual material - power point presentation (fall detection system) or video (fall scenarios)	Single interview (s)	Single interview (s) WITH video (fall scenarios)	Questionnaire/s	Idea Generation & Concept Development	Device (Re-) Design & Prototype Development	Prototype Testing in-House & Trials in Real Field	Deployment in the Market & User Feedback
Abbate et al (2012)	N.a.		N.a.								X							X		
Ariani et al (2010)	X			X															X	
Barralon et al (2013)		X		X															X	
Bloch et al (2011)		X			X														X	
Bourke et al (2008a)	X			X															X	
Bourke et al (2007)	X			X															X	
Bourke et al (2012)	X									X								X		
		X		X															X	
Bourke et al (2010a)	X	X		X															X	
Bourke et al (2010b)	X	X		X															X	
Bourke et al (2008b)		X				X													X	
Bourke et al (2010c)	X	X		X															X	

Author(s) and year	Involvement of older people																			
	Focus of involvement																Stages of involvement*[4]			
	Technical aspects										Views of older people						I	II	III	IV
	Simulation / performance																			
			Setting																	
			Lab	Real-life																
	Scripted ADL*[3]	Everyday life activities		Home	Hospital	Nursing home	Community centre	Independent living facility	Long -term care facility	N.s. clearly	Focus group interview (s) WITH prototype	Focus group interview (s)	Focus group interview (s) WITH visual material - power point presentation (fall detection system) or video (fall scenarios)	Single interview (s)	Single interview (s) WITH video (fall scenarios)	Questionnaire/s	Idea Generation & Concept Development	Device (Re-) Design & Prototype Development	Prototype Testing in-House & Trials in Real Field	Deployment in the Market & User Feedback
Bourke et al (2008c)		X				X													X	
Boyle & Karunanithi (2008)		X			X														X	
Campo et al (2010)		X				X													X	
Che-Chang et al (2007)	X									X								X		
De la Guia Solaz et al (2010)	X			X															X	
Demiris et al (2004)	N.a.		N.a.									X					X			
Fourty et al (2009)		X								X			X					X	X	
Marquis-Faulkes et al (2005)	N.a.		N.a.										X					X		
Gietzelt et al (2012)		X		X										X					X	
Godfrey et al (2011)	X			X															X	
Goevercin et al (2010)	N.a.		N.a.										X			X	X			
Holzinger et al (2010)	N.a.		N.a.								X					X		X		

Author(s) and year	Involvement of older people																			
	Focus of involvement																Stages of involvement*[4]			
	Technical aspects										Views of older people						I	II	III	IV
	Simulation / performance																			
			Setting																	
			Lab	Real-life																
	Scripted ADL*[3]	Everyday life activities		Home	Hospital	Nursing home	Community centre	Independent living facility	Long -term care facility	N.s. clearly	Focus group interview (s) WITH prototype	Focus group interview (s)	Focus group interview (s) WITH visual material - power point presentation (fall detection system) or video (fall scenarios)	Single interview (s)	Single interview (s) WITH video (fall scenarios)	Questionnaire/s	Idea Generation & Concept Development	Device (Re-) Design & Prototype Development	Prototype Testing in-House & Trials in Real Field	Deployment in the Market & User Feedback
Horton (2008)	N.a.		N.a.											X						X
Huang et al (2012)	X									X								X		
Jantaraprim et al (2012)	X									X								X		
Kangas et al (2012)		X				X													X	
Kangas et al (2009)	X					X													X	
Kerdegari et al (2012)	X		X															X		
Lai et al (2010)	X		X															X		
Lai et al (2011)	X		X															X		
Lindemann et al (2005)	X									X									X	
Shinmoto et al (2013)	X				X											X			X	
Liu & Lockhart (2013)	X		X															X		
Londei et al (2009)	N.a.		N.a.												X	X		X		
McKenna et al (2006)	N.a.		N.a.									X	X					X		

Author(s) and year	Involvement of older people																			
	Focus of involvement																Stages of involvement*[4]			
	Technical aspects										Views of older people						I	II	III	IV
	Simulation / performance																			
			Setting																	
			Lab	Real-life																
	Scripted ADL*[3]	Everyday life activities		Home	Hospital	Nursing home	Community centre	Independent living facility	Long -term care facility	N.s. clearly	Focus group interview (s) WITH prototype	Focus group interview (s)	Focus group interview (s) WITH visual material - power point presentation (fall detection system) or video (fall scenarios)	Single interview (s)	Single interview (s) WITH video (fall scenarios)	Questionnaire/s	Idea Generation & Concept Development	Device (Re-) Design & Prototype Development	Prototype Testing in-House & Trials in Real Field	Deployment in the Market & User Feedback
Yu et al (2013)	X									X				X				X		
Narasimhan (2012)	X						X											X		
Parker et al (2008)	N.a.		N.a.													X	X			
Quagliarella et al (2008a)	X									X								X		
Quagliarella et al (2008b)	X			X															X	
Rantz et al (2013)	X	X	X					X								X		X	X	
Sixsmith & Johnson (2004)	N.a.		N.a.										X					X		
Soaz et al (2012)		X								X									X	
Stone & Skubic (2014)		X						X											X	
Tamrat et al (2012)	X	X					X	X	X										X	
van de Ven et al (2008a)		X			X														X	
van de Ven et al (2008b)		X							X										X	
Wang et al		X								X									X	

Author(s) and year	Involvement of older people																			
	**Focus of involvement**																**Stages of involvement*[4]**			
	**Technical aspects**										**Views of older people**						**I**	**II**	**III**	**IV**
	Simulation / performance																			
			Setting																	
			Lab	Real-life																
	Scripted ADL*[3]	Everyday life activities		Home	Hospital	Nursing home	Community centre	Independent living facility	Long -term care facility	N.s. clearly	Focus group interview (s) WITH prototype	Focus group interview (s)	Focus group interview (s) WITH visual material - power point presentation (fall detection system) or video (fall scenarios)	Single interview (s)	Single interview (s) WITH video (fall scenarios)	Questionnaire/s	Idea Generation & Concept Development	Device (Re-) Design & Prototype Development	Prototype Testing in-House & Trials in Real Field	Deployment in the Market & User Feedback
(2014)																				
Wu & Xue (2010)	X									X								X		
Wu & Xue (2008)	X									X								X		
Zhang et al (2006)	X		X																X	
John et al (2008)		X		X															X	

*[3]Activity of daily living; *[4]according to Shah et al[33]
